# Vape Club: Exploring Non-Profit Regulatory Models for the Supply of Vaporised Nicotine Products

**DOI:** 10.3390/ijerph15081744

**Published:** 2018-08-14

**Authors:** Coral Gartner, Marilyn Bromberg, Tanya Musgrove, Kathy Luong

**Affiliations:** 1 School of Public Health, Faculty of Medicine, The University of Queensland, Brisbane 4006, Australia; kathy.luong@uq.net.au; 2 Queensland Alliance for Environmental Health Sciences, Faculty of Health and Behavioural Sciences, The University of Queensland, Brisbane 4072, Australia; 3 Law School, University of Western Australia, Perth 6009, Australia; marilyn.bromberg@uwa.edu.au (M.B.); tanya.musgrove@uwa.edu.au (T.M.)

**Keywords:** e-cigarettes, nicotine, non-profit model, cannabis social club, medical cannabis, kava, tobacco

## Abstract

Vaporised nicotine products (VNPs) that are not approved as therapeutic goods are banned in some countries, including Australia, Singapore, and Thailand. We reviewed two non-profit regulatory options, private clubs and the Australian Therapeutic Goods Administration Special Access Scheme (SAS) that have been applied to other controlled substances (such as cannabis) as a potential model for regulating VNPs as an alternative to prohibition. The legal status of private cannabis clubs varies between the United States, Canada, Belgium, Spain, and Uruguay. Legal frameworks exist for cannabis clubs in some countries, but most operate in a legal grey area. Kava social clubs existed in the Northern Territory, Australia, until the federal government banned importation of kava. Access to medical cannabis in Australia is allowed as an unapproved therapeutic good via the SAS. In Australia, the SAS Category C appears to be the most feasible option to widen access to VNPs, but it may have limited acceptability to vapers and smokers. The private club model would require new legislation but could be potentially more acceptable if clubs were permitted to operate outside a medical framework. Consumer and regulator support for these models is currently unknown. Without similar restrictions applied to smoked tobacco products, these models may have only a limited impact on smoking prevalence. Further research could explore whether these models could be options for regulating smoked tobacco products.

## 1. Introduction

Vaporised nicotine products (VNPs), also known as e-cigarettes, have presented a regulatory challenge to policy makers because they do not neatly fit the existing regulatory frameworks for nicotine products [1]. Medicinal nicotine products (e.g., nicotine patches) must meet high manufacturing quality standards, demonstrate efficacy in randomised controlled trials, and be marketed conservatively as short-term cessation aids. Attributes such as addictiveness and pleasure are minimised. In contrast, tobacco products have few mandated quality standards and regulations aim to deter use rather than to ensure product quality. VNPs that do not contain tobacco share some characteristics of medicinal products (e.g., can assist smoking cessation) and some characteristics of tobacco products (e.g., marketed as consumer products).

Regulation of VNPs varies between countries and is an evolving process. They are regulated as tobacco, medicines, consumer products, or some combination of these [2]. Most Western democracies (e.g., UK, USA, Europe) follow a dual-track regulatory system, which allows VNPs to be sold either as consumer/tobacco products with some restrictions on sales and promotion, or as medicines if therapeutic claims are made [3].

Some countries (Australia and Malaysia) will only allow VNPs to be sold if they are approved as medicines [2]. This restriction is effectively a ban for nicotine-containing vaping products. For example, Australia regulates VNPs as either prescription-only medicines, if for therapeutic use, or as dangerous poisons, if for non-therapeutic use [4]. Because dangerous poisons are not allowed to be supplied for domestic use, VNPs cannot be sold as non-therapeutic products. Since no VNPs are approved as therapeutic goods in Australia, they are effectively prohibited for sale, possession, or use, apart from some limited exceptions, which allow Australians to access unapproved therapeutic goods under certain conditions [5]. Theoretically, it is possible that a VNP may be approved as a therapeutic good at some point in the future. However, so far, there have only been two VNPs (one is an e-cigarette, the other is a pressurised aerosol device) that have received medicinal approval and neither of these were commercialised [3]. Most countries that have imposed medicines-only regulation on VNPs have changed or are in the process of changing their policy to dual-track regulation to allow VNPs to be sold either as a consumer product, or a therapeutic good if approved as a cessation aid. These countries include Sweden [6], Norway [7], Switzerland [8], Denmark [9], Canada [10], and New Zealand [11]. However, recent attempts to allow VNPs to also be sold as consumer goods in Australia have been unsuccessful [12,13]. In March 2018, an Australian Parliamentary Inquiry into the Use and Marketing of Electronic Cigarettes and Personal Vaporisers in Australia concluded a majority report that recommended no change in current laws [14]. Furthermore, Australia’s Federal Health Minister stated in late 2017 that he would never change current Australian laws to allow VNPs to be sold as consumer products [15]. Other countries have completely banned the sale, and in some cases, possession and use of all vaping products, including those that do not contain nicotine. Examples include Singapore [16], Thailand [2], and one state of Australia (Western Australia) has banned sale of vaporising devices regardless of whether they contain nicotine or not (nicotine-free vaping products can be sold in all other Australian States and Territories) [17].

Consequently, VNPs are treated much like illicit drugs in some countries. Penalties for possession of VNPs in Singapore include fines up to $10,000 SGD and/or imprisonment for up to 6 months [16]. The penalties are twice as much for repeat offenders [16]. The UK foreign travel advice for Thailand warns that possession of a vaporiser device can lead to fines or imprisonment for up to ten years, stating that, ‘several British Nationals have been arrested for possession of vaporisers and e-cigarettes’ [18]. Similarly, some Australian vapers have been prosecuted for possession of VNPs [19]. The maximum penalty for possession or use of nicotine in non-therapeutic products (other than tobacco intended for smoking) varies between Australian states and territories ranging from fines of $1100 (New South Wales) to $45,000 (Western Australia) AUD [20]. Three jurisdictions include the possibility of imprisonment for up to 12 months (Northern Territory) [21] or two years (Tasmania and Australian Capital Territory) [22,23]. Despite these penalties, many Australians access VNPs illegally [24].

Given the ongoing strong opposition to allowing VNPs to be sold as general consumer products in some countries, such as Australia, Singapore and Thailand, we explored alternative regulatory approaches that could facilitate access for addicted smokers who might benefit from the switch from the traditional method of smoking to vaping. We consider two non-profit models that have been applied to two controlled substances (cannabis and kava) as a potential model for VNP regulation in countries that prohibit their sale, possession, and use: (1) compassionate/social clubs and (2) the Australian Therapeutic Goods Administration’s Special Access Scheme (SAS).

## 2. Cannabis Social Clubs, Kava Clubs and Other Medical Cannabis Regulation

Table 1 summarises the legislation that applies to the operation of cannabis clubs in various countries (USA, Canada, Spain, Belgium, Uruguay), kava clubs, and the SAS for Medical Cannabis in Australia.

### 2.1. Cannabis Social Clubs (CSC)

Cannabis social clubs (CSCs) operate in a number of countries, including in North and South America, Canada, and Europe. The first cannabis social club (CSC) was created in San Francisco, California [25]. Each individual CSC operates differently, depending on the jurisdiction where they are located and even within jurisdictions, this may vary. However, they are usually non-profit organisations that are funded through members’ donations or membership fees [26]. CSCs involve members meeting at a private location where they can purchase and use cannabis while encouraging members to create a supportive network for each other [25,27,28]. The clubs were developed initially to improve accessibility to cannabis for patients who previously reported difficulty obtaining it and who could not grow their own [26]. CSCs may provide additional services, including those focusing on harm-reduction and mental health, to assist club members [25].

With some exceptions, CSCs generally operate illegally or within a legal grey area. In the past, select Canadian jurisdictions (Vancouver and Victoria) permitted a license to be obtained in order to operate a CSC to supply cannabis, but federal laws may still render them illegal. Health Canada had threatened to raid CSCs for selling or advertising cannabis [49]. The police had also charged people with drug offences due to their involvement with CSCs in Canada [50,51]. However, most charges were dropped or discharged so that these clubs seemingly operated without interference by the local government as a tolerated illegal activity [28]. However, as of July 2018, Canada’s legislation changed regarding the distribution, production, and sale of cannabis for recreational use. Under this new legislation, the drug is available for purchase and consumption for reasons other than medical purposes under The Cannabis Act [52].

CSCs in Spain rely on the notion that the possession and distribution of cannabis would not constitute an offence under the criminal code, so long as the operation occurs solely for personal use within a closed circuit of adults [36]. This code only bans commercial sale and production, and possession and use in public places [36]. While all possession, cultivation, and distribution/trade of cannabis is prohibited in Belgium [36], the possession of cannabis in amounts considered to be for personal use (e.g., 3 g or one cultivated plant) is tolerated if there are no aggravating factors, such as violence or public order disturbance [38]. Belgian CSCs argue that by maintaining their distribution to no more than one plant per member, they operate within the scope of personal use and are within the lowest priority for prosecution. It is unclear whether this argument is accepted by legal authorities [38].

In Uruguay, cannabis use is legal for registered users over the age of 18 and regulated by the government [36]. The Institute for the Regulation and Control of Cannabis (IRCCA) authorises CSCs to operate. CSCs must also register as non-profit organisations with the Registry Office at the Ministry of Education and Culture [36]. Registration and authorisation involves meeting an extensive list of legal requirements. The legal status of CSCs allows for consistent regulations throughout Uruguay and leaves little room for self-regulation. In addition to CSCs, adults can obtain cannabis legally by self-growing or through pharmacies. However, individuals are not permitted to access cannabis through more than one method, that is, via any combination of these routes. Additionally, an individual cannot be a member of more than one CSC at any given time and there are restrictions on membership sizes and the quantity and potency of cannabis permitted [39]. These restrictions are to ensure that individuals obtain no more than 40 g per month for personal use [36]. CSCs in Uruguay are not allowed to advertise their establishments because the law bans the promotion of cannabis products. Taxes are collected from sales to fund public health campaigns [40].

### 2.2. Kava Social Clubs

Kava comes from the root of a plant called Piper methysticum. In the early 1980s, missionaries introduced kava to Aboriginal people in northern Australia as an alternative to alcohol [44]. The Northern Territory Government passed the Kava Management Act 1998 (NT) [45] because people were selling kava at expensive prices on the black market, leaving some families of kava drinkers without enough money to buy food [46]. The Act made it legal to possess kava in less than trafficable quantities, but the supply, manufacture, or production of kava without a licence was illegal [45]. In Western Arnhem Land, a kava social club was developed where the operation funded sport, recreational, and traditional activities [47]. Communities developed guidelines to regulate kava sale and consumption. License applicants were required to disclose the intended kava selling price and their suggested use of profits to support education, community benefit, and development. Additionally, restricted trading hours were implemented, and community-based services strictly monitored heavy kava consumers [47]. In 2007, the federal government banned the commercial importation of kava (except for medical and research purposes) and ceased issuing permits for personal use, which ended legal kava sales and dismantled the Northern Territory licensing system [48].

### 2.3. Authorised Supply of Unregistered Therapeutic Goods, e.g., Australian TGA Special Access Scheme

Some countries allow health practitioners to supply unregistered therapeutic goods to patients on an individual basis as part of the patient’s medical treatment. For example, New Zealand’s *Medicines Act 1981* (S29) allows unregistered medicines to be supplied to, and administered by, medical practitioners for patient treatment without needing to justify the use of the unapproved medicine. In Australia, the TGA Special Access Scheme (SAS) allows health practitioners to import and/or supply an unapproved therapeutic good to a single patient on a case-by-case basis depending upon whether the patient falls under one of the following three categories. Category A covers persons who are seriously ill with a condition and in whom death is reasonably likely to occur within a matter of months, or from which premature death is likely to occur in the absence of early treatment [53]. Category B covers all other patients and requires the prescriber to apply to the TGA for the use of the product [41]. Under this category, registered medical practitioners apply to the Scheme for their patient to access medical cannabis. The application must include a justification for needing the product, in addition to providing efficacy and safety information [54]. Category C is a new notification scheme. This category allows health practitioners to supply unapproved therapeutic goods that are listed within the Rules for the indications listed and by the type of health practitioner listed, without requiring prior approval. These goods are deemed to have an established history of use [54].

## 3. Application of Non-Profit Club or SAS Regulatory Models to VNPs

Non-profit clubs for cannabis and kava, and the SAS for medicines (including medical cannabis), have developed in response to demand for access to substances that are not currently available in a country via existing regulatory frameworks for consumer goods or medicines. Hence, they may have relevance for VNPs in countries where VNPs are not available but where demand for these products exists. There are additional similarities between using cannabis and VNPs that may make it appropriate to apply the CSC model to VNPs. People may face social stigma as a result of using cannabis and vaping is also increasingly being stigmatised in some countries. People who use cannabis have their own culture, as do people who vape. These similarities support the idea that it could be helpful for vapers to go to a location with like-minded people who use the same products.

While some overseas jurisdictions have specific legislation covering CSCs (Uruguay, Victoria, and Vancouver in Canada, which is relevant until the new *Cannabis Act* comes into effect), many operate within a legal ‘grey area’ or as tolerated illegal activities. If vape clubs were established in countries that currently treat VNPs like illicit drugs, it would be crucial that they are legalised, so that vapers could visit them without fear of prosecution. Some jurisdictions could potentially grant authorisation via the current mechanisms for approving the sale, possession, and use of ‘dangerous poisons’ [55]. The necessary level of regulatory oversight would require careful consideration and could range from self-regulation up to a closely controlled government-licensing system. Examples of self-regulation include the Code of Conduct for European Cannabis Social Clubs published by the European Coalition for Just and Effective Drug Policy (ENCOD) [36], which provides guidelines for operating CSCs for the non-profit cultivation and distribution of cannabis to its members for their own personal use [37]. Examples of government-licensed models include CSCs in Vancouver and Victoria in Canada (until the legalisation of cannabis comes into effect) and Uruguay, and the previous regulation of kava in Australia.

Regulations could aim to minimise potential diversion of products to outside of the club membership. Some aspects of the previous regulations for kava management, such as limits on the quantity permitted for personal use may be appropriate. Some CSCs provide only a location to consume cannabis, while others supply cannabis and allow it to be used onsite. There are even CSCs that only supply the cannabis and prohibit use on the premises. As most vapers use nicotine throughout the day, regulation would need to allow club members to also use VNPs in locations other than the club facilities.

The SAS model uses the same regulatory framework that applies to medicines but does not require the products to be formally approved by the medicines regulator. Thus, the SAS model removes the main barrier to supply of VNPs within countries that enforce mandatory medicines regulation of VNPs, namely the lack of any approved products. As such, it may be a more viable option in Australia in the short-term where the framework already exists. However, the complexity of the Category B process is likely to be too onerous to be practicable for VNPs. A simpler option would be to list nicotine for inhalation for the treatment of nicotine dependence on the Category C list [53].

### Benefits and Limitations of Non-Profit Models

The potential benefits and limitations of the club and SAS models for regulating VNPs are summarised in Table 2.

The primary benefit of these non-profit models is that they could provide a pathway for smokers to switch to VNPs in countries that currently have no or only limited options for access. Because the private club model generally operates on a non-profit basis, this may relieve some concerns about commercial retailing of VNPs, such as advertising and promotion, and commercial incentives to recruit non-smoking youth. However, the possibility exists that young non-smoking adults may also set up vape clubs in addition to groups of smokers who want to quit smoking. In this respect, the SAS may have an advantage as it includes a health practitioner as a gatekeeper which reduces the risk of supply of VNPs to individuals who are not already using nicotine, such as young non-smokers. None of the cannabis club models we reviewed stated that a doctor or pharmacist was required to write a prescription or approve a person entry to the club. Although, if legislation was passed to make vape clubs legal, confirmation that applicants are already using nicotine either by vaping or smoking before approving their membership could be a requirement.

Vape clubs could provide novice vapers with a network of experienced vapers to support them as they transition from smoking to vaping, which they may not have access to if they simply purchased VNPs online or from a general retailer without specialist vaping knowledge. This support network may be particularly valuable for smokers who experience difficulties adjusting to vaping. Admittedly, vapers can easily and quickly seek advice on VNPs online and by word-of-mouth, without needing to attend a private club [56]. Furthermore, specialist vape shops can also provide this type of support, as they already do in countries that allow retail sales of vaping products [57].

Vape clubs could assist vapers to access VNPs at reduced cost as supplies could be purchased in bulk quantities for distribution to all club members, allowing volume discounts to be negotiated with suppliers. Having access to VNPs at a lower cost than cigarettes would provide an incentive for smokers to switch from smoking to vaping [58].

The main limitation of both options (whether private club or SAS) is that they are much more restrictive compared to the ways in which VNPs are regulated in most high income countries, or compared to how tobacco cigarettes are sold. They would have a smaller impact on smoking prevalence than allowing general retail sales of VNPs because cigarettes would remain much more accessible than VNPs unless similar restrictions were also applied to smoked tobacco products (STPs). Without widespread advertising, many smokers may remain unaware of how to access VNPs through these avenues. An option could be to allow vape clubs to promote their services on social media as well as to healthcare professionals in order to increase the awareness of their services. In countries that allow retailing of nicotine-free vaping products, vape shops would likely also refer customers who want to vape nicotine to a club or the SAS.

The need to introduce a new regulatory framework is another major barrier to establishing a vape club model or SAS in countries where this framework does not currently exist. Careful consideration is needed as to whether setting up such a framework is a good investment or whether other regulatory options should be pursued. In this regard, the SAS Category C route appears to be the most straightforward option to implement in Australia as the legal framework already exists. However, implementing this option may delay implementing another, potentially more appropriate regulatory model.

The quality of products supplied via vape clubs could be highly variable unless the government required minimum standards for the products that could be supplied. This contrasts to VNPs supplied through the SAS that would be expected to meet pharmaceutical quality standards. Regulations apply to VNPs in many countries that allow their sale (e.g., United Kingdom) [59]. Similar regulations could apply to VNPs supplied via vape clubs.

The acceptability of these models to smokers and vapers is unknown. Similar to cannabis and kava, which can be used both therapeutically and recreationally, the boundary between therapeutic and recreational use of VNPs is indistinct. Putative recreational use of VNPs may provide a therapeutic benefit if it helps maintain abstinence from smoking. However, many vapers do not see themselves as having an illness and often reject the medicalisation of VNPs [60]. These vapers view VNPs as a less harmful alternative to smoking rather than a medical intervention. Hence, a private club model may not have widespread acceptance if it is seen as being part of a medical framework. While not all CSCs operate solely to supply cannabis for medical purposes, the club model has its roots in compassionate access, while the SAS is explicitly a medical model and VNPs supplied via the SAS would be treated as therapeutic goods. Nonetheless, many vapers report using VNPs for the purpose of quitting smoking or preventing relapse to smoking [61], that is, as an unapproved smoking cessation aid or a form of maintenance therapy. The use of VNPs is self-directed; the user administers and monitors their own consumption of nicotine [62]. However, by encouraging patterns of use that are associated with smoking cessation (e.g., daily use), these strategies have the possibility of increasing continued abstinence from smoking and reduce the prevalence of smoking overall. A compassionate club model may have greater acceptability to smokers who have difficulty quitting smoking using existing approved methods, but who also accept a medicalised approach to smoking cessation. Current compassionate clubs existing in Canada are able to secure a reliable supply of their product through a trusted relationship with their cultivator to ensure product safety and quality [28]. As such, research into the acceptability of a private club model among vapers and smokers would be needed to determine the likely success or failure of the model, which may also be dependent on the level of regulation applied to how the clubs could operate.

The SAS model could provide more consistent access than a private club model as smokers and vapers living in remote areas might be less likely to have access to a vape club. Since the club model depends on vapers creating and managing the clubs, there may be many vapers who would be interested in using the services of a vape club, but do not have the time or resources to start one. Some priority populations of smokers, such as individuals with a mental illness could benefit from switching from smoking to vaping, but may find joining a private club a barrier to access.

A limitation of these models is that they do not adequately accommodate temporary visitors to the country, such as tourists who vape. It would not be practical to require tourists to join a vape club in order to be permitted to possess and use VNPs while in the country. In this respect, the SAS Category C may be more viable since tourists could visit a medical practitioner to obtain a prescription to authorise their possession and use of VNPs. This method would still place an additional burden and expense on international tourists. Unless most doctors were willing to prescribe VNPs, it could be impractical way to obtain access. Obtaining the VNPs would also be problematic unless many pharmacies maintained a supply or if tourists were allowed to bring their own VNPs with them (which would require them to obtain a prescription prior to their arrival).

## 4. Application of Non-Profit Club or SAS Regulatory Models to Smoked Tobacco Products (STPs)

While we have primarily reviewed the vape club and SAS models in terms of applicability to VNPs, these models could also be applied to STPs, if governments took the step of abolishing the sale of STPs [63]. Under such a scenario, providing a non-profit option (run by either individuals or a corporate entity with non-profit status) for smokers who are unable to quit smoking and are not able to grow their own tobacco could reduce black-market sales. Others have also discussed the benefits of transferring the supply of STPs to non-profit enterprises [64,65]. Private non-profit clubs could provide an additional model to consider as part of the current discussions about tobacco endgame strategies. Further research could explore applying this regulatory model in the context of STPs.

## 5. Conclusions

We explored whether two different non-profit models (the private club model and the Australian TGA SAS) could be used to provide access to VNPs for smokers who could benefit from switching to a less harmful alternative to cigarettes in countries that currently ban their sale, possession, and use. In Australia, the SAS Category C route appears to be the most straightforward option to implement in the short-term as the legal framework already exists. However, the SAS offers only marginal benefits over existing pathways for accessing unapproved therapeutic goods and may be unacceptable to many vapers and smokers who oppose the medicalisation of VNPs. Therefore, a private club model may potentially be more acceptable to vapers, if vape clubs are established as adult social clubs rather than ‘compassionate clubs’. However, the club model also has many limitations including the need for new legislation to protect members from prosecution, and unknown acceptability to vapers, smokers, and regulators. Furthermore, neither of these models are ideal for distributing VNPs if the goal is to compete with, and replace, STPs unless similar restrictions were also placed on STPs. Thus, their impact on smoking prevalence may be limited unless regulation of both VNPs and STPs are considered and reformed in tandem.

## Figures and Tables

**Table 1:** Examples of the regulatory models (social club and special access scheme) for access to controlled substances (Cannabis and Kava).

Model	Brief Description	Non-Profit	Legal Status (e.g., Established Clearly in Law, Unclear/Grey, Illegal But Tolerated)	Registration/Documentation/Approvals Required (e.g., Club Membership Documents, Medical Prescription, Club Registration)	Restrictions		
					Age of Participant	Diagnoses	Quantity of Substance
Cannabis Clubs: USA [25,26,27]	Supported by membership fees/donations	√	Illegal but tolerated, users who have a medical marijuana ID card typically will not be prosecuted	Medical marijuana ID card	State-dependent	State-dependent All States will give a medical marijuana card if the user has HIV/AIDs	State-dependent
Compassionate Cannabis Clubs: Canada [28,29,30,31,32,33,34,35]	Places where people who are normally very ill and require medical marijuana can purchase safe marijuana	√	The Canadian Federal Government passed the Cannabis Act which will come into force on 17 October 2018. This law legalises cannabis for recreational use. Until this Act comes into force, it is illegal to use cannabis for recreational use nationwide (except Vancouver and Victoria, BC). Some clubs outside these cities were raided.	License	Vancouver, BC: 19 years of age Victoria, BC: 19 years of age	Unknown	Unknown
Cannabis Social Club**:** Spain [36,37]	Private clubs for the non-commercial distribution of cannabis	√	Non-criminal but not legalised. Most follow ENCOD code of conduct	Registration as a not for profit association with National Registry of Organisations	18 or 21+ years of age	Not required	60–90 g per month
Cannabis Social Club: Belgium [36,38]	Private clubs for the non-commercial distribution of cannabis	√	Illegal but tolerated	Registration as a not for profit association with National Registry of Organisations	18 or 21+ years of age	Required for CSCs operating solely for medical users, otherwise not required	10–30 g per month
Cannabis Social Club: Uruguay [36,39,40]	One of three legal ways for individuals to obtain cannabis	√	Legal and regulated	Registration as a non-profit association with the registry and IRCCA	18+ years of age	Accepted but not a requirement	40 g per month
Australian Special Access Scheme for Medicinal Cannabis [41,42,43]	Registered doctors with Australian Register of Therapeutic Goods apply on a case by case basis for patient to be able to import cannabis or else procure it through an Australian manufacturer	√	Legal and regulated	Medical doctor must be registered and apply for their patient to access the cannabis. The patient will also need to apply for permission to the relevant authority in their State/Territory	Unknown	None	Determined by medical doctor
Kava: Northern Territory, Australia [44,45,46,47,48]	Legal to possess a small amount of kava. Licences previously issued to sell kava by retail or wholesale (to licensed retailers)		Importation banned apart for medical and research purposes.	Can possess a specific quantity. Before importation ban, licences were issued to manufacture and sell kava.	18 years of age	None	Possess less than two kilograms, or less than four kava plants (but not in drinkable form)

Laws covering cannabis in Australia, Canada, USA, Spain, Belgium and Uruguay and kava in Australia were identified by searching several legal and political databases (including LexisNexis AU, AGIS, Westlaw and the University of Western Australia’s search engine entitled Onesearch) with keywords including ‘cannabis’, ‘cannabis laws’, ‘marijuana laws’, ‘kava management act’ and ‘kava licensing’.

**Table 2:** Benefits and limitations of social club and special access scheme models.

Model	Benefits	Limitations
Not-for-profit social club	Provides legal access to VNPs for harm reduction Reduces commercial incentive to recruit young non-smokers, including risk of aggressive marketing practices Provides a supportive environment to assist smokers to learn about vaping May achieve lower prices for members through bulk purchases Could operate alongside a commercial retail market for nicotine-free vaporiser products Can operate under a medical or a recreational framework Could provide a model for access to smoked tobacco products if retail sales abolished	Would require new laws to ensure members are not prosecuted—risk of overly burdensome regulation More restrictive than current access to smoked tobacco products May not be accessible to the most disadvantaged smokers (living in remote areas, people with mental illness) May not be acceptable to vapers/smokers—e.g., could be viewed as a medical model (Compassion clubs) Difficult for smokers to become aware of the clubs without advertising Products purchased may be of variable quality Problematic for international visitors to access Likely to have little impact on smoking prevalence
Approval to supply domestically as unapproved therapeutic good e.g., TGA Special Access Scheme (Category C)	Existing regulatory framework in some countries (Australia) Allows access to products without relying on a manufacturer applying for medicines approval Health practitioner as gatekeeper reduces risk of young non-smokers Quality of products supplied may be higher than for social club model (e.g., meet pharmaceutical standards). Provides legal access to VNPs for harm reduction	Maintains medical model which has low acceptability among vapers More restrictive than current access to smoked tobacco products May require new laws in some countries Expensive due to involvement of health practitioners (e.g., medical practitioner, pharmacist) Health practitioners may not be willing to take responsibility for prescribing/supplying unapproved therapeutic goods Problematic for international visitors to access Likely to have little impact on smoking prevalence

VNP Vaporised Nicotine Product.
